# Child- and parent-related determinants for out-of-home care in a nationwide population with neurodevelopmental disorders: a register-based Finnish birth cohort 1997 study

**DOI:** 10.1007/s00787-024-02406-w

**Published:** 2024-03-02

**Authors:** Sanni Penttilä, Mika Niemelä, Helinä Hakko, Markus Keski-Säntti, Tiina Ristikari, Sami Räsänen

**Affiliations:** 1 Faculty of Medicine, Research Unit of Clinical Medicine, University of Oulu, Oulu, Finland; 2 Faculty of Medicine, Research Unit of Population Health, University of Oulu, Oulu, Finland; 3 Department of Psychiatry, Oulu University Hospital, Oulu, Finland; 4 The Finnish Institute for Health and Welfare (THL), Helsinki, Finland; 5 ITLA Children’s Foundation, Helsinki, Finland

**Keywords:** Out of home care, Neuro developmental disorder, ADHD, Psychiatric disorder

## Abstract

Neurodevelopmental disorders (NDDs) are among the most common health issues in childhood and adolescence. Psychiatric disorders are known to be overrepresented among children using child welfare services and placed in out-of-home care (OHC). Child- and parent-related determinants for OHC among a national population with NDDs were evaluated utilising longitudinal register data from the national Finnish Birth Cohort 1997 (*n* = 58,802) from birth to 18 years (1997–2015). The cohort members with NDDs (*n* = 5,143, 9% of total cohort) formed our study population. Based on their history of OHC, cohort members with NDD were categorised to OHC (*n* = 903) and non-OHC groups (*n* = 4,240). Of all cohort members with NDDs, 17.6% had a history of OHC. Within NDDs, a significant excess of ADHD diagnosis was observed in the OHC group compared to the non-OHC group (49% vs. 26%). The OHC group with NDDs was significantly characterised by having comorbid psychiatric diagnosis for conduct and oppositional disorders (adj. RR 2.21), substance use disorders (adj. RR 1.61) and depression and anxiety disorders (adj. RR 1.60). Of all parent-related determinants, the most prevailing in the OHC group compared to the non-OHC group, was social assistance received by parent (88% vs. 44.5%). The longer the period (in years) for received social assistance, the greater the likelihood for OHC (adj. RRs range from 2.41 for one year to 5.24 for over 4 years). Further, significantly associating determinants for OHC were parental psychiatric disorders (adj. RR 1.42) and parental death (adj. RR 1.23). Our findings from the population-based cohort of children and adolescents with NDDs highlight the importance of screening and assessment of family situation. Also, effective prevention and treating of comorbid psychiatric disorders, especially conduct and oppositional disorders is essential.

**Supplementary Information:**

The online version contains supplementary material available at 10.1007/s00787-024-02406-w.

## Introduction

Neurodevelopmental disorders (NDDs) consist of a group of conditions with a clinically wide range of neurocognitive deficits and psychiatric problems. These disorders include attention deficit hyperactivity disorder (ADHD), autism spectrum disorders (ASD), tic disorders, intellectual disability, and specific learning, motor, and communication disorders (ICD-10 and DSM-5). They are characterised by early childhood onset and often lifelong symptoms that are dimensional and trait-like in nature. The overlap of core neurodevelopmental and neurocognitive symptoms between different NDDs is common and psychiatric comorbidities are frequently observed. NDDs with their typical psychiatric comorbidities, such as anxiety and oppositional and conduct disorders, are known to cause lifelong challenges in health, education, and social life [1–3].

Epidemiological data that include comprehensive evaluation about the prevalence of NDDs is relatively rare; however, these disorders are regarded as very common in the general population. A national study from the US reported a combined prevalence of 17% for ten developmental disabilities in US children aged 3–17 years [4]. In addition, many studies concerning the prevalence of separate NDDs such as ADHD, ASD and learning disabilities indicate that NDDs form a significant group of disorders [5–7].

NDDs have also been shown to be highly over-represented among children who receive child welfare services and, especially, among those who are placed in out-of-home care (OHC) [8–12]. Recently, based on a nationwide Finnish birth cohort population, it was reported that the prevalence of NDDs in children and adolescents placed in OHC was more than 3 times higher compared to those never placed in OHC (26.1% vs. 7.2%) [9].

Internationally, the number of children placed in OHC is very high. It is estimated to be over 2.7 million worldwide [13], almost 760,000 in European Union countries, and approximately 105,000 in the United Kingdom [14]. Furthermore, it is assessed that in western countries, 3–6% of all children are placed in OHC during their childhood [15, 16]. According to a Finnish study of all children born in 1997, 5.7% of the population had a history of OHC placement before age 18 [9]. The high numbers of children in OHC and the prevalence of NDDs among these children indicate that they form a significant population with social issues and health-related challenges.

The observation that NDDs appear and could be diagnosed already in childhood provides an opportunity to target early-phase preventive actions in health care at children with NDDs [3]. By recognising the most common determinants associating with institutionalised service use in this population it would be possible to find ways to prevent the need of last-resort child welfare services. However, the individual- and family-related determinants elevating the risk for OHC among the population of children with NDD have not been in the focus of intensive research. Earlier literature recognises the association of family-related determinants, such as parental mental health problems, substance use, and low income with OHC [15, 17–19]. Socioeconomic determinants, together with cultural orientation in the communities (such as individualism and collectivism), seem to indicate OHC placement rates [20]. Franzen et al. (2008) analysed a large Swedish birth cohort register data set and found that cumulation of certain socio-economic risk factors, such as parental low education, unemployment and received social assistance, increased the risk for OHC placement dramatically [21]. However, there is a research gap in studies addressing comprehensively the impact of individual- and parent-related determinants in association with NDDs and OHC placement of children and adolescents at the population level.

In the current study, we were able to explore child- and parent-related determinants for OHC in a nationwide child and adolescent population with NDDs. We had access to longitudinal register data of all Finnish Birth Cohort 1997 (FBC1997) members who had been diagnosed with NDD in special health services before age 18. The child- and parent-related determinants under study comprised social and educational factors, morbidity, health care service use, rehabilitative service use, and psychiatric comorbidities. Based on the findings of previous literature about epidemiology and associating determinants of OHC and neurodevelopmental disorders, we formulated two hypotheses:

### Hypothesis 1

We hypothesised that the population with NDDs and OHC placements has increased psychiatric comorbidity and therefore has also used more psychiatric and other health services, when compared to the population with NDDs without OHC placements.

### Hypothesis 2

We hypothesised that adverse parent-related determinants such as received social assistance, low education, parental substance use disorders and mental health problems are more common in the population with NDDs and OHC placements when compared to the population with NDDs without OHC placements.

To our knowledge, there are no previous studies including both child- and parent-related determinants for OHC assessed in a nationwide population of children and adolescents with NDDs. The Finnish health and social care service system represents the quality of the welfare states in the Nordic region with a strong emphasis on equality, accessibility, and quality for the whole population. We assume that the results of our study are applicable and relevant for the health and social service context in the majority of western countries.

## Materials and methods

### Study population and data sources

The data source of this study was register data from the national Finnish Birth Cohort 1997 (FBC 1997) administered by the Finnish Institute on Health and Welfare (THL). This birth cohort includes register-based data of all children who were born in Finland in 1997 and who survived the perinatal period (*n* = 58,802). The FBC 1997 data include data from several national registers such as the national Care Register for Health Care (CRHC) and the Child Welfare Register (CWR). The cohort was followed from birth to 18th birthday during the years 1997–2015. The FBC 1997 data also contain register data of the biological parents of these children. The official Finnish personal identity codes enable linkage of data from different registers. Those who emigrated or died before age 18 were excluded. After exclusion, *n* = 57,152.

### Neurodevelopmental disorders

All birth cohort members with any diagnosis of a neurodevelopmental disorder before age 18 were extracted (*n* = 5,143, 9.0%) from the CRHC maintained by the THL. Data on diagnosis includes all diagnoses set in special health care services. The neurodevelopmental diagnoses, based on the 10th revision of the International Classification of Diseases (ICD-10), covered intellectual disabilities (F70.x-F79.x), disorders of speech and language (F80.xx), specific developmental disorders of scholastic skills (F81.xx), motor disorders (F82), mixed specific developmental disorders (F83), autism and pervasive developmental disorders (F84.xx), disturbance of activity and attention and hyperkinetic disorders (F90.xx), tic disorders (F95.xx) and other specified behavioural and emotional disorders with onset usually occurring in childhood and adolescence (F98.9). Primary and secondary diagnoses were checked to identify persons with NDDs. A person may have had a diagnosis belonging to several diagnostic categories. (Statistics of OHC group in Appendix A Table A1).

### Out-of-home care

Out-of-home care (OHC) status was collected from the Child Welfare Register (CWR), which is maintained by the THL. It includes national register data on the start and end dates of out-of-home placements, placement settings, and legal grounds of the placement decision from 1991 onwards. Those cohort members diagnosed with NDD who had a history of any placement in OHC before age 18 were included in the OHC group. Of the total subpopulation of the cohort members with NDDs (*n* = 5,143), 903 (17.6%) had a history of OHC. The rest of the cohort members who had NDD but had never been placed in OHC formed the non-OHC-group (*n* = 4,240).

### Child-related determinants

For each cohort member with diagnosis of a neurodevelopmental disorder (*n* = 5,143), following ICD-10 based comorbid diagnostic groups for psychiatric disorders were analysed: Conduct and oppositional disorders, psychotic and bipolar disorders, depression and anxiety disorders, FAS, substance-related disorders, self-harm and suicidality and PTSD.

Other child-related determinants included health service use such as inpatient and outpatient visits to psychiatric clinics and child neurology, external causes of hospitalisations, psychotropic purchases, disability allowance and rehabilitation. Inpatient and outpatient visits to child neurology, child and adolescent psychiatry and psychiatry were searched from the CRHC. The CRHC was also utilised to collect information on unintentional injuries and self-injury and their external causes such as accidents, suicidality and poisonings that can cause acute hospitalisations and are known to associate with poor mental health [22, 23]. In addition, information on psychotropic purchases, disability allowance and rehabilitation financed by the Social Insurance Institution of Finland were collected from the register of the Social Insurance Institution of Finland (KELA).

### Parent-related determinants

Data on biological parents of the cohort members with NDDs included 5,143 mothers and 5,064 fathers. The current study utilised the data of parent age at birth of cohort member (The Finnish Population Information System), highest education (Statistics Finland), amount and duration of social assistance (THL), disability pension (KELA), diagnosis for parental psychiatric disorders (CRHC, ICD10 all F-codes), diagnosis for parental neurological disorders (CRHC, ICD10 all G-codes), parental brain injury (CRHC, ICD10 S06), and parental death (Statistic Finland). All these variables were followed until the cohort member reached the age of 18, apart from education, which was measured at the end of 2014 due to data restrictions. Data on living arrangements of the family (separated during follow-up, living together during follow-up, child placed when under 1 year old) were searched from the Finnish Population Information System and CWR.

### Data analysis

The risk ratios (RRs) with 95% confidence intervals (CIs) were computed to quantify the association between placement in OHC and all analysed determinants. Adjusted risk ratios for out-of-home care (OHC) placement by child- and parent-related determinants were reported by multiple modified Poisson regression analyses using robust standard errors (HC3) [24]. Poisson regression analysis included model 1 for child-related determinants, model 2 for parent-related determinants, and model 3 for combined child- and parent-related determinants. Of the child-related determinants, psychiatric comorbidities were included in model 1 and 3 considering the high correlation of them with those for service use, psychotropic purchases, and rehabilitation. FAS and PTSD were excluded due to small n values. All parental-related determinants except living arrangements were included in model 2 and 3. Statistical analysis was conducted with R 4.1.1. [25].

## Results

**Fig. 1:**
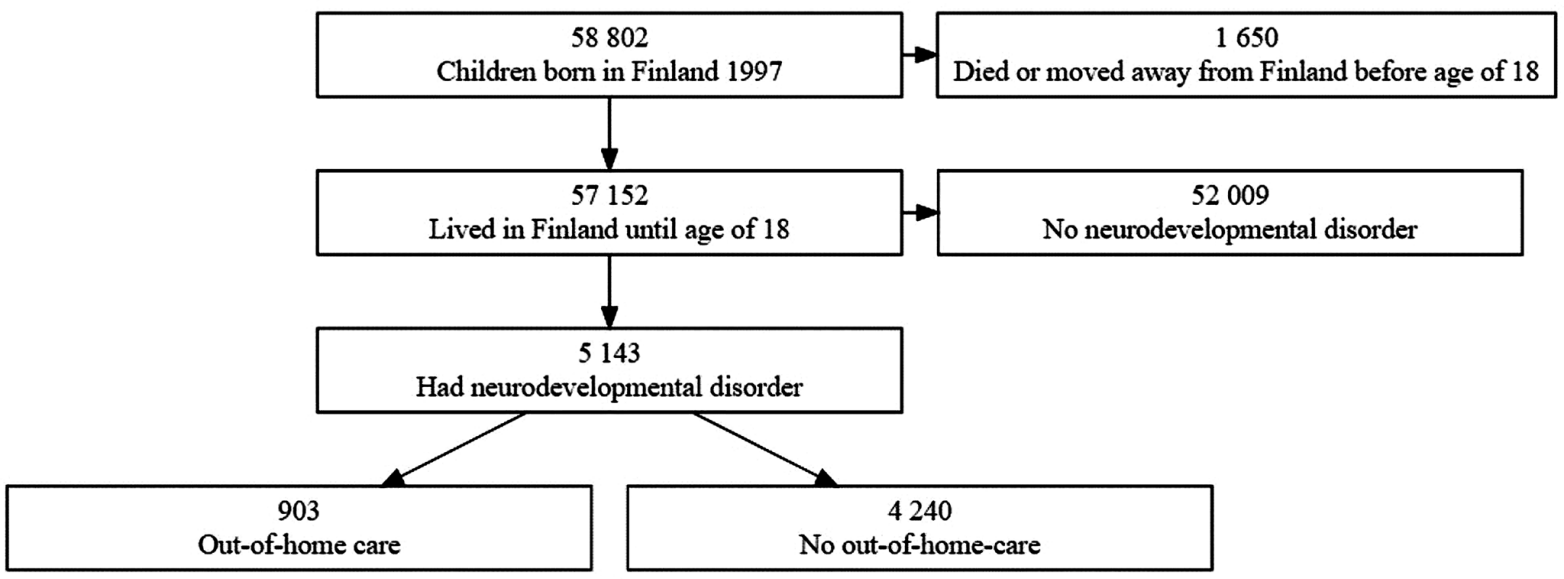
Flowchart for study population

Of all Finnish Birth Cohort 1997 (FBC 1997) individuals who lived in Finland up to age of 18 years (*n* = 57,152), 9.0% (*n* = 5,143) had at least one diagnosis of NDDs assessed in special health care. Of these cohort members with NDD, 17.6% (*n* = 903) had a history of out-of-home care placement (OHC). Study population is described in Fig. 1.

### Prevalence of neurodevelopmental disorders

**Fig. 2:**
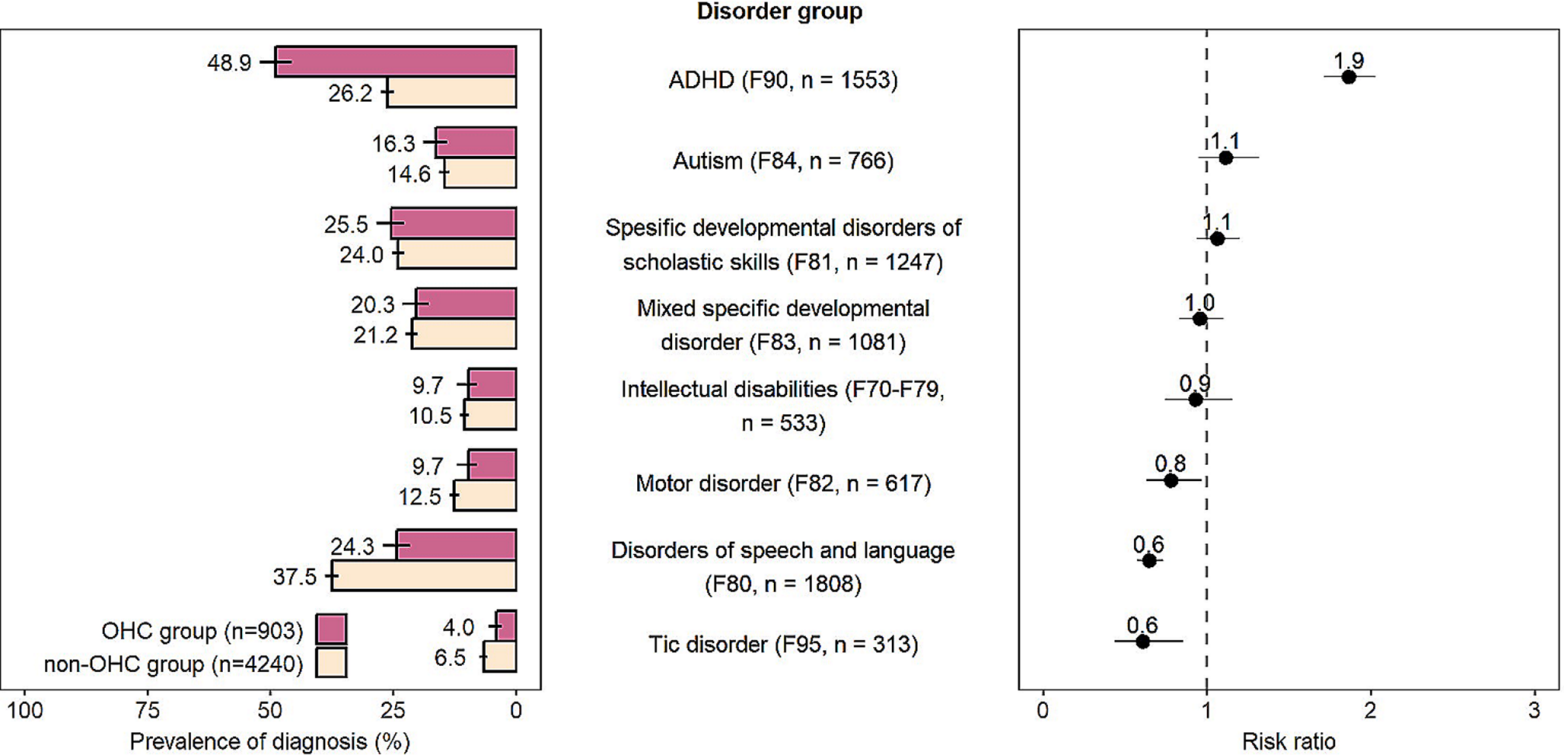
Diagnostic distribution of the Finnish Birth Cohort 1997 members with neurodevelopmental disorders, by out-of-home care (OHC) status

As Fig. 2 shows, the prevalence of ADHD was almost double in the OHC group compared to the non-OHC group (48.9% vs. 26.2%, RR 1.9). Furthermore, compared to the non-OHC group, the OHC group was less likely to have a diagnosis for motor disorders (9.7% vs. 12.5%, RR 0.8), disorders of speech and language (24.3% vs. 37.5%, RR 0.6) and tic disorder (4.0% vs. 6.5%, RR 0.6).

Of the cohort members with diagnosed NDDs, 17.6% had an OHC placement history. The mean age at first diagnosis was 9.48 years and the mean age at first placement was 10.32 years (Supplemental Table A1). Additional analysis in the subsample of NDD patients with OHC placements showed that 52.6% of them had received their first NDD diagnosis before their first OHC placement. In those whose first NDD diagnosis preceded the first OHC placement, the mean time-interval between NDD diagnosis and OHC placement was 5.5 years. Correspondingly, this time interval was 4.3 years when the OHC placement preceded the assessment of NDD diagnosis (Supplemental Table A1).

### Psychiatric comorbidities

**Fig. 3:**
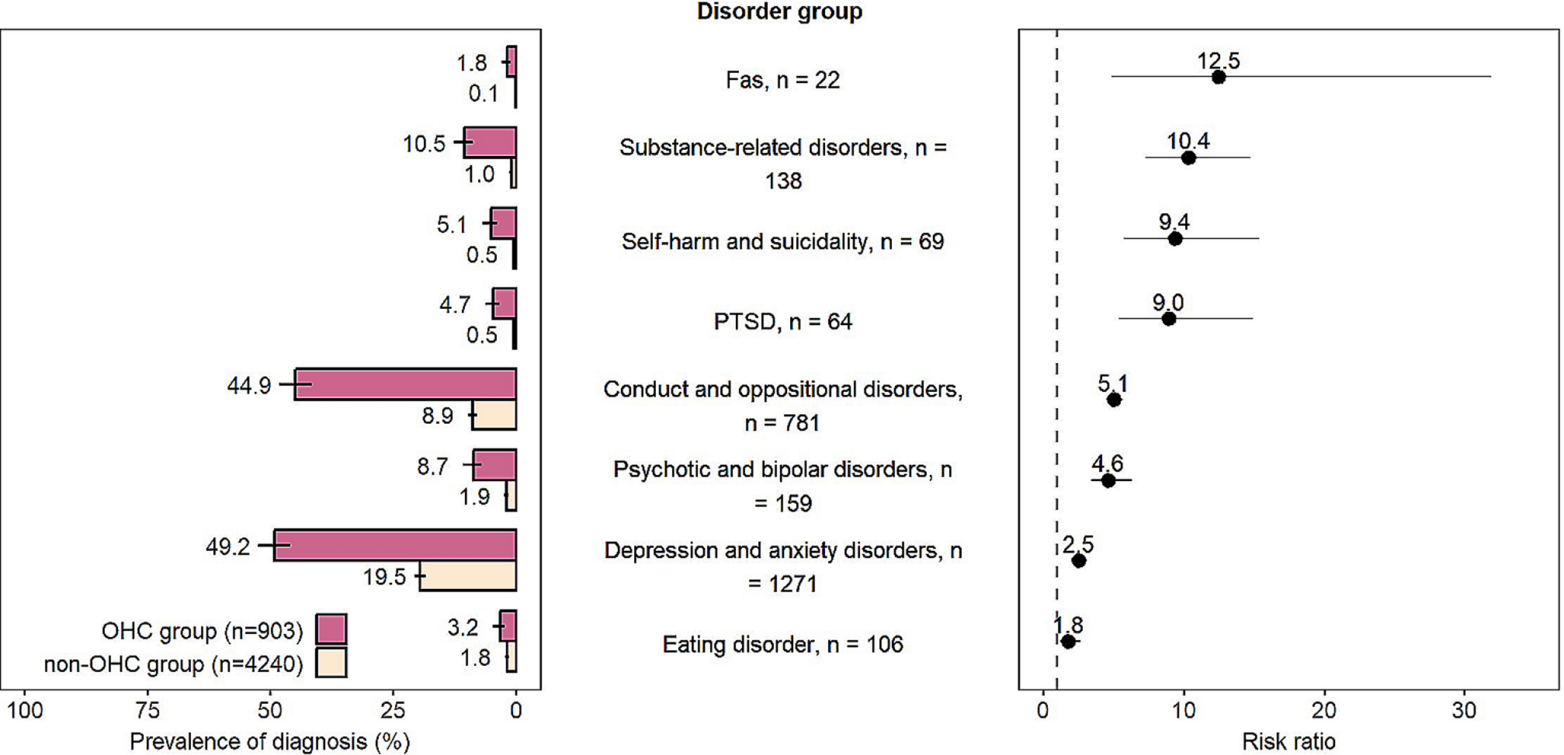
Psychiatric comorbidities of the Finnish Birth Cohort 1997 members with neurodevelopmental disorders, by out-of-home care (OHC) status

Figure 3 presents the comorbid psychiatric disorders of the study population with neurodevelopmental disorders. Those in the OHC group were 10.4 times more likely to have a comorbid substance-related disorder compared to the non-OHC group (10.5% vs. 1.0%, RR 10.4). Similarly, the OHC group was characterised by having significantly more often FAS (1.8% vs. 0.1%, RR 12.5), self-harm and suicidality (5.1% vs. 0.5%, RR 9.4), conduct and oppositional disorders (44.9% vs. 8.9%, RR 5.1), psychotic and bipolar disorders (8.7% vs. 1.9%, RR 4.6), depression and anxiety disorders (49.2% vs. 19.5%, RR 2.5), and eating disorder (3.2% vs. 1.8%, RR 1.8).

Additional analysis showed that 30.3% (*n* = 274) of cohort members with NDDs in the OHC group had a combination of ADHD and conduct and oppositional disorders, accounting for 65.9% of those with ADHD diagnosis. Vice versa, in the OHC group, 67.7% of those who had been diagnosed with conduct and oppositional disorders had ADHD. Respectively, in the non-OHC group, 5.9% (*n* = 250) had a combination of ADHD and conduct and oppositional disorders.

### Other child-related determinants

In general, as seen in Table 1, health service use as child-related determinant was more prevalent in the OHC group than in the non-OHC group. The OHC and non-OHC groups differed statistically significantly in specialised level health service use, including adolescent psychiatry (71.5% vs. 25.6%, RR 2.8), psychiatry (19.3% vs. 6.5%, RR 3.0) and child psychiatry (58.5% vs. 27.9%, RR 2.1). The OHC group also had more commonly utilised psychiatric inpatient care (1.8% vs. 0.2%, RR 10.7), adolescent psychiatric inpatient care (25.0% vs. 4.5%, RR 5.6) and child psychiatric inpatient care (22.4% vs. 4.9%, RR 4.6%). The OHC group was also more likely to have prescribed psychotropic purchases (69.1% vs. 38.51%. RR 1.8) than the non-OHC group. In terms of the use of rehabilitation services, the OHC group had more often received rehabilitative psychotherapy (1% vs. 0.2%, RR 4.2) and rehabilitative allowance (30.2% vs. 19.5%, RR 1.6) than the non-OHC group. In addition, the OHC group was less likely to have received disability allowance before the age of 6 compared to the non-OHC group (16.9% vs. 24.7%, RR 0.7).

**Table 1:** Service use of the Finnish Birth Cohort 1997 members with neurodevelopmental disorders, by out-of-home care (OHC) status

Service	OHC (*n* = 903)	non-OHC (*n* = 4240)	Risk ratio (RR) with 95% CI
**Specialised level psychiatric and child neurology health care. n (%)**			
Child neurology, outpatient Child neurology, inpatient Child psychiatry, outpatient Child psychiatry, inpatient Adolescent psychiatry, outpatient Adolescent psychiatry, inpatient Psychiatry, outpatient Psychiatry, inpatient	551 (61.0%) 176 (19.5%) 375 (58.5%) 202 (22.4%) 646 (71.5%) 207 (25.0%) 174 (19.3%) 16 (1.8%)	2738 (64.6%) 922 (21.8%) 1183 (27.9%) 207 (4.9%) 1084 (25.6%) 191 (4.5%) 276 (6.5%) 7 (0.2%)	0.9 (0.89–1) 0.9 (0.78–1.04) 2.1 (1.95–2.25) 4.6 (3.83–5.49) 2.8 (2.62–2.99) 5.6 (4.43–26.01) 3.0 (2.48–3.53) 10.7 (4.43–6.65)
**Prescribed psychotropic purchase, n (%)**	279 (69.1%)	1633 (38.5%)	1.8 (1.69–1.9)
**Rehabilitation services (KELA), n (%)**			
Vocational rehabilitation Discretional rehabilitation Intensive medical rehabilitation Rehabilitation Rehabilitative psychotherapy	103 (11.4%) 46 (5.1%) 127 (14.1%) 228 (25.3%) 9 (1%)	437 (10.3%) 195 (4.6%) 690 (16.3%) 1084 (25.6%) 10 (0.2%)	1.1 (0.9–1.35) 1.1 (0.81–1.51) 0.9 (0.73–1.03) 1.0 (0.87–1.12) 4.2 (1.72–10.37)
**Rehabilitation allowance (16–18 years), n (%)**	273 (30.2%)	826 (19.5%)	1.6 (1.38–1.74)
**Disability allowance, n (%)**			
No disability allowance Before age of 6 As 6-17-year-old	482 (53.4%) 153 (16.9%) 268 (29.7%)	2304 (54.3%) 1045 (24.7%) 891 (21.0%)	1.0 (0.92–1.05)
			0.7 (0.59–0.80)
			1.4 (1.26–1.59)

**Fig. 4:**
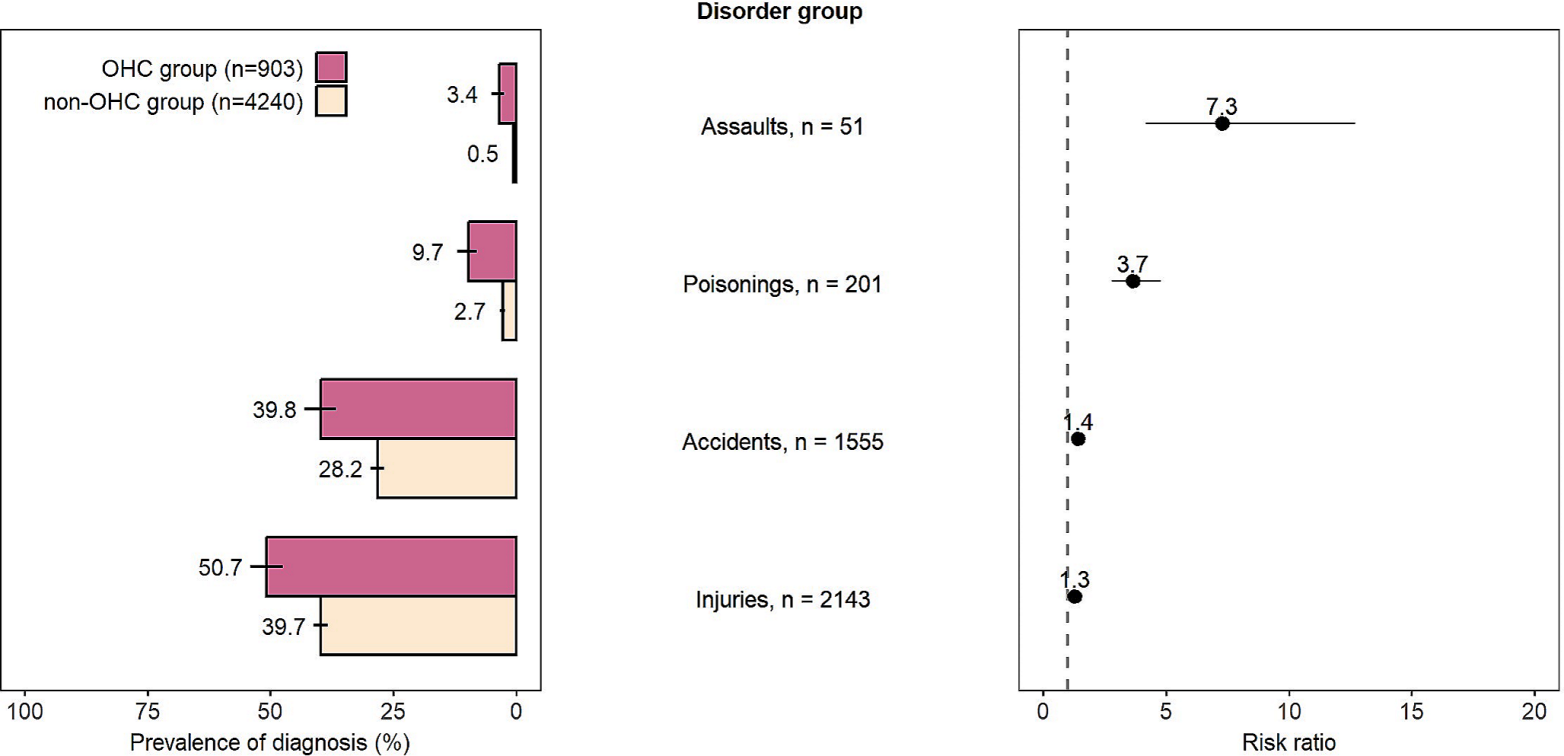
External causes for hospitalisation of the Finnish Birth Cohort 1997 members with neurodevelopmental disorders, by out-of-home care (OHC) status

External causes for hospitalisation are presented in Fig. 4 (For ICD-codes, see Appendix A Table A2). The OHC group was more likely to have a hospital visit due to an assault (3.4% vs. 0.5%, RR 7.3) or poisoning (9.7% vs. 2.7%, RR 3.7). The OHC group was also more likely to have an accident-related visit (39.8% vs. 28.2%, RR 1.4) and visits relating to injuries (50.7% vs. 39.7%, RR 1.3).

### Parent-related determinants

Table 2 presents the parent-related determinants of the study population. A higher proportion of parents in the OHC group had no secondary education compared to the non-OHC group (17.3% vs. 4.6%, RR 3.8). Furthermore, the parents of the OHC group were more likely to have disability pension (30.6% vs. 10.3%, RR 3.0) and had received social assistance for more than 4 years more often (58.6% vs. 13.2%, RR 4.4) than the parents of the non-OHC group. In the OHC group, families who did not receive social assistance were less common (11.9% vs. 55.5%, RR 0.2) compared to the non-OHC group. Parental death was more common in the OHC group than in the non-OHC group (13.2% vs. 4.4%, RR 3.0). In addition, a higher proportion of parents in the OHC group, compared to the non-OHC group, had a diagnosis of psychiatric disorders (67.8% vs. 31.6%, RR 2.1), neurological diseases (42.4% vs. 37.5%, RR 1.1), or a diagnosis for brain injury (14.8% vs. 6.8%, RR 2.2).

Biological parents of the OHC group were younger than those in the non-OHC group (mother’s age at birth (mean) 27.9 years vs. 29.8 years, *p* < 0.001, father’s age at birth (mean) 31.3. years vs. 32.4 years, *p* < 0.001).

**Table 2:** Parent-related determinants of the Finnish Birth Cohort 1997 members with neurodevelopmental disorders, by out-of-home care (OHC) status

Parent-related determinants	OHC (*n* = 903)	non-OHC (*n* = 4240)	Risk ratio (RR) with 95% CI
Separated	663 (73.4%)	1825 (43.0%)	1.7 (1.62–1.8)
Together	155 (17.2%)	2415 (57.0%)	0.3 (0.26–0.35)
Child placed when under 1 year old	85 (9.4%)	-	-
**Parental highest education, n (%)**			
No secondary	156 (17.3%)	195 (4.6%)	3.8 (3.08–4.58)
Secondary	549 (60.8%)	160 (17.7%)	38 (4.2%)
Lower tertiary	1857 (43.8%)	1533 (36.2%)	655 (15.5%)
Upper tertiary or higher	1.4 (1.3–1.48)	0.5 (0.42–0.57)	0.3 (0.2–0.37)
**Parental disability pension, n (%)**	276 (30.6%)	437 (10.3%)	2.97 (2.6–3.39)
**Social assistance, n (%)**			
No	107 (11.9%)	2354 (55.5%)	0.2 (0.18–0.26)
1 year	127 (14.1%)	799 (18.8%)	0.7 (0.63–0.89)
2 years	61 (6.8%)	274 (6.5%)	1.0 (0.8–1.37)
4 years	79 (8.8%)	255 (6.0%)	1.5 (1.14–1.85)
Over 4 years	529 (58.6%)	558 (13.2%)	4.4 (4.05–4.89)
**Parental death, n (%)**	119 (13.2%)	188 (4.4%)	3.0 (2.39–3.7)
**Parental psychiatric diagnosis, n (%)**	291 (67.8%)	1340 (31.6%)	2.1 (2.01–2.28)
**Parental neurologic diagnosis, n (%)**	383 (42.4%)	1589 (37.5%)	1.1 (1.04–1.23)
**Parental brain injury, n (%)**	134 (14.8%)	289 (6.8%)	2.2 (1.8–2.64)

### Multiple modified Poisson regression analyses for out-of-home-care placement by child and parent -related determinants

Table 3 presents the results of multiple modified Poisson regression analysis for OHC by child- and parent-related determinants. In model 1 for child-related determinants, statistically significant association for placement to OHC was found in all psychiatric comorbidities except for eating disorder of the child. In model 2 for parent-related determinants, the increased likelihood for placement to OHC of the offspring was associated with parents receiving social assistance, parental death and parental psychiatric diagnosis, while decreased likelihood for out-of-home placement was related to higher level of education of parents. In combined model 3 including both child- and parent-related determinants, the statistically significant associations with OHC remained mainly the same, expect for self-harm of the child.

**Table 3:** Adjusted risk ratios and confidence intervals from multiple modified Poisson regression using robust standard errors (HC3) for out-of-home care (OHC) placement by child- and parent-related determinants

Variable	Model 1: Child-related determinants (Adjusted RR, 95% CI)	Model 2: Parent-related determinants (Adjusted RR, 95% CI)	Model 3: Combined determinants (Adjusted RR, 95% CI)
**Child-related determinants**			
Conduct and oppositional disorder	3.25 (2.86–3.68)		2.21 (1.97–2.49)
Depression and anxiety disorders	1.85 (1.63–2.1)		1.60 (1.42–1.8)
Psychosis and bipolar disorders	1.42 (1.18–1.7)		1.24 (1.03–1.51)
Substance use disorder	1.98 (1.69–2.32)		1.61 (1.35–1.92)
Self-harm	1.54 (1.24–1.91)		1.16 (0.91–1.48)
Eating disorder	1.15 (0.85–1.55)		1.13 (0.84–1.51)
**Parent-related determinants**			
Parents highest education (ref. no edu) Upper tertiary or higher			
		0.49 (0.35–0.69)	0.51 (0.37–0.71)
Lower tertiary		0.60 (0.5–0.72)	0.66 (0.55–0.79)
Secondary		0.82 (0.72–0.93)	0.84 (0.74–0.96)
Parental disability pension		1.10 (0.98–1.23)	1.09 (0.97–1.22)
Social assistance (ref. no)			
Social assistance – 1 year		2.55 (1.98–3.28)	2.41 (1.90–3.06)
Social assistance – 2 years		3.15 (2.34–4.25)	2.79 (2.10–3.71)
Social assistance – 4 years		3.71 (2.81–4.9)	3.15 (2.4–4.12)
Social assistance – Over 4 years		6.63 (5.32–8.26)	5.24 (4.21–6.52)
Parental death		1.28 (1.11–1.47)	1.24 (1.07–1.44)
Parental psychiatric or substance use diagnosis		1.68 (1.46–1.92)	1.42 (1.25–1.62)
Parental brain injury		1.10 (0.96–1.25)	1.07 (0.93–1.23)
Parental neurological diagnosis		0.91 (0.81–1.0)	0.93 (0.83–1.03)

## Discussion

Neurodevelopmental disorders (NDDs) are among the most common health issues in the child and adolescent general population [4]. These disorders are known to associate with various life-time health, social and educational adversities [3, 16, 26]. NDDs are also known to be markedly overrepresented among children and adolescents who have a history of out-of-home care (OHC), which is the uttermost child welfare intervention [8–11]. To the best of our knowledge, there is no comprehensive research information of the determinants that associate with OHC in population with NDDs. Research-based information is needed to develop more effective preventive and rehabilitative strategies and methods.

The current study analysed social and health care register data of the nationwide Finnish Birth Cohort 1997 (FBC1997) population with diagnosed NDDs to explore diversely child- and parent-related determinants that are associated with OHC status. The data in our analyses covered the age period from birth to 18 years of the cohort members. We believe that this is the first study evaluating child- and parent-related determinants associating with OHC from longitudinal multi-register data from a nationwide birth cohort including all children and adolescents with NDDs diagnosed in special health care.

The main finding of this study was that parent-related determinants were the most essential factors associating with OHC status among the child and adolescent population with diagnosed NDDs. Of these determinants, the prevalence of social assistance received by parent (88% vs. 44.5%), parents living separately (73.4% vs. 43.0%), and psychiatric disorders of parent (67.8% vs. 31.6%) was twice as high in the OHC group than in the non-OHC group. In the multiple regression analysis, long duration of received social assistance was emphasised as the most strongly associating determinant for the OHC status of the offspring. The longer the period of received social assistance the more toxic it seems to be for the offspring. We assume that long period for social assistance reflects prolonged stressors and prolonged need for diverse support in the family. Our hypothesis which assumed that adverse parent-related determinants are more common in the population with NDDs and OHC placements was supported by these results. Earlier studies have shown that the above-mentioned parent-related factors, as well as low education, have generally been associated with child welfare service use and the OHC status of the offspring [15, 17–19]. In general, worse economic situation in the family is known to associate with different adverse consequences of children. Peverill et al. have reported that lower socio-economic status of the family is in association with higher levels of psychopathology in general [27]. In addition, earlier studies have indicated that family poverty has an association with NDDs such as ADHD in children [28]. Our findings confirm the importance of the family environment and parents’ situation for the wellbeing of children, especially among children and adolescent with NDDs. Our study as well as earlier research around the topic highlights the need of rehabilitative approaches focusing on the whole family. For example, the use of integrated care models that aim to develop patient-centred and coordinated service chains to social and health care services for children and families [29–31]. Also, an earlier Finnish study concerning service pathways to child psychiatric inpatient care showed that clinical practices are too narrowly focused on the child and his/her problems as an individual [32]. In practice, the results of our study point out that, when recognising neurodevelopmental symptoms in a child, appropriate assessment of the social situation of the family and screening of parent’s mental health should also be performed. According to Takalo et al. 2022, solving adverse family situations requires multidisciplinary collaboration. This kind of collaboration is effective when utilising structured models to which professionals are committed across sectoral boundaries [33].

This study also reveals the heavy burden of psychiatric disorders in the OHC group with NDDs. As we assumed in our first hypothesis, psychiatric comorbidity was significantly more common in the OHC group compared to the non-OHC group. In the multiple regression analysis, comorbid conduct and oppositional disorders had the highest association with OHC status among child-related determinants. In the OHC group, depression and anxiety disorders were more than twice as common (49.2% vs. 19.5%) and conduct and oppositional disorders about five times more common (44.9% vs. 8.9%). The OHC group used more commonly specialised treatment in child and adolescence psychiatry, which is presumable observing the commonness of the comorbid psychiatric disorders. They also used more often psychotropic medication. In addition, suicidality/self-harm and substance use disorders treated in special health care were at remarkably higher level in the OHC group. These findings are in line with earlier studies showing that psychiatric disorders and psychiatric comorbidities are known to be typical with neurodevelopmental disorders [3], especially among children placed in OHC [9, 11, 16]. This study advocates efforts to create effective treatment models for children and adolescents in OHC. Treatment-based foster care or therapeutic foster care could offer ways to improve mental health and other critical outcomes of this vulnerable population [34–36].

We were also able to explore the distribution of different diagnoses within NDDs and compare them between the OHC and the non-OHC groups. Diagnosis for ADHD was twice as prevalent in the OHC group compared to the non-OHC group. It was noteworthy that approximately half of the cohort members with NDDs in the OHC group had received an ADHD diagnosis. This finding confirms the previous studies that have reported that especially ADHD and ADHD-like symptoms are common among children using child welfare services [8].

Additional analyses showed that a combination of ADHD and oppositional and conduct disorder diagnosis was found in a third of the cohort members in the OHC group. Furthermore, in the OHC group, two thirds of those with an ADHD diagnosis had a comorbid oppositional or conduct disorder diagnosis. These findings are in line with previous studies suggesting that ADHD with comorbid oppositional and conduct disorders may specifically contribute to social difficulties and poor psychosocial functioning in child and adolescent populations [2, 37, 38]. Prevention and treatment of comorbid oppositional and conduct disorders seem to be crucial elements in the population with NDDs.

In our study, disability allowance was less commonly received in the OHC group before the age of 6 years. The disability allowance rate signals that the use of rehabilitative services was less common before the age of 6 years in the OHC group compared to the non-OHC group. This result might indicate that early rehabilitation could be a protective and preventive trajectory for OHC. Lower use of rehabilitative services might be a consequence of adverse family situation. This assumption is justified by earlier studies that have reported parents’ decreased possibilities to ensure child’s healthcare and rehabilitation services in stressful family situations [28].

In the subsample of NDD patients with OHC-history, about half of the OHC group had their first NDD diagnosis before their first placement in OHC and the mean time-interval between NDD diagnosis and OHC placements was 5.5 years. The corresponding time interval was 4.3 years in those whose NDD diagnosis was set after the first OHC placement. A detailed inspection of the timing and the quality of health services use and the characteristics of the OHC placements in the OHC group and the subgroups in it is an essential issue for future research. There may be service gaps and neglected health needs among these children, as well as health service use that does not respond to the real needs of these children and the families of children with diagnosed NDDs.

### Strengths and limitations

A strength of this study was the possibility to analyse national birth cohort longitudinal follow-up data from several national registers from birth to age 18. The present study combined longitudinal register data including the whole national birth cohort population with register data of their biological parents. This gave the opportunity to scrutinise data on individual and family level. Public health care services in Finland cover the whole population. In Finland, child health clinics follow the health, growth, and development of all children. Early rehabilitation as well as guidance to social services and special health services are equally available for every child and family. The register data did not include diagnoses from primary health care and private health care, which is why disorders with milder symptoms may not be included in this study. The data used in this study may therefore underestimate the frequency of neurodevelopmental disorders and their comorbidities as well as psychiatric and substance use disorders and other health conditions of the parents. Otherwise, the CRHR data concerning diagnosis can be regarded comprehensive and reliable [39, 40].

A limitation of the current study was that temporal evaluation of different determinants and outcomes was not considered. Temporal evaluation between neurodevelopmental disorders and OHC and associating child- and family-related determinants is an essential topic for further research. Gender differences and age dispersion of neurodevelopmental and other psychiatric disorders were not evaluated, either. Furthermore, the present study did not include an accurate evaluation of multimorbidity and different combinations of comorbidities, which could be a topic for further research. In a register study, it is not possible to consider qualitative determinants like maltreatment, exposure to violence, or quality of parenting. Kaiser et al. 2011 note that parenting and developmental problems share a complex relationship, and that parenting could be a significant mediator between ADHD severity and child outcomes [41].

## Conclusions

The present study addresses that in the population with NDDs, parent-related determinants are notably linked with OHC placements of children and adolescents. For that reason, when diagnosing and treating ADHD and other NDDs in children in health care, careful attention should be paid to family level adversities, such as parental mental health problems and family poverty. Therefore, family resources should be routinely checked in child health care services and the requisite family interventions should be arranged in addition to the child’s health care.

The high prevalence of comorbid psychiatric disorders underlines the importance of evaluating and treating possible comorbidities when diagnosing and treating children with neurodevelopmental disorders. This study emphasises the necessity of targeting effective interventions for common psychiatric comorbidities, especially depression and anxiety disorders as well as conduct and oppositional disorders, to children and adolescents with a diagnosis of NDDs. Psychiatric and mental health interventions that are integrated into child welfare and foster care service systems are needed.

The study provides evidence about significant specialised health service use and heavy burden of psychiatric disorders in the study population. Preventive strategies and multidisciplinary service models are needed. Interdisciplinary studies combining social and health data are needed to develop and implement effective, integrated, and preventive services targeted to vulnerable high-need and high-cost populations of children and adolescents. Children with NDD diagnosis and OHC status are most evidently one of those populations.

In the future research, a multivariate approach and temporal analyses are warranted for investigating more deeply the association between NDD and OHC and related determinants. For example, this should include analyses of preplacement factors of OHC that influence the social and health outcomes of children and adolescents.

## Electronic supplementary material

Below is the link to the electronic supplementary material.

Supplementary Material 1

## Data Availability

The data collected in the Finnish Birth Cohort 1997 will only be processed by the THL research group, who have been granted permission to process the data.

## References

[1] Joelsson P, Chudal R, Gyllenberg D, Kesti AK, Hinkka-Yli-Salomäki S, Virtanen JP et al (2016) Demographic characteristics and Psychiatric Comorbidity of children and adolescents diagnosed with ADHD in Specialized Healthcare. Child Psychiatry Hum Dev 47(4):574–582 26399420 10.1007/s10578-015-0591-6

[2] Steinhausen HC (2009) The heterogeneity of causes and courses of attention-deficit/hyperactivity disorder. Acta Psychiatr Scand 120(5):392–399 19807721 10.1111/j.1600-0447.2009.01446.x

[3] Thapar A, Cooper M, Rutter M (2017) Neurodevelopmental disorders. Lancet Psychiatry 4(4):339–346 27979720 10.1016/S2215-0366(16)30376-5

[4] Zablotsky B, Black LI, Maenner MJ, Schieve LA, Danielson ML, Bitsko RH et al (2019) Prevalence and Trends of Developmental Disabilities among children in the United States: 2009–2017. Pediatrics 144(4):e20190811 31558576 10.1542/peds.2019-0811 PMC7076808

[5] Dalsgaard S, Thorsteinsson E, Trabjerg BB, Schullehner J, Plana-Ripoll O, Brikell I et al (2020) Incidence rates and cumulative incidences of the full spectrum of diagnosed Mental disorders in Childhood and Adolescence. JAMA Psychiatry 77(2):155–164 31746968 10.1001/jamapsychiatry.2019.3523 PMC6902162

[6] Erskine HE, Baxter AJ, Patton G, Moffitt TE, Patel V, Whiteford HA et al (2017) The global coverage of prevalence data for mental disorders in children and adolescents. Epidemiol Psychiatr Sci 26(4):395–402 26786507 10.1017/S2045796015001158 PMC6998634

[7] Arrhenius B, Gyllenberg D, Chudal R, Lehti V, Sucksdorff M, Sourander O et al (2018) Social risk factors for speech, scholastic and coordination disorders: a nationwide register-based study. BMC Public Health 18(1):739 29902994 10.1186/s12889-018-5650-z PMC6002992

[8] Klein B, Damiani-Taraba G, Koster A, Campbell J, Scholz C (2015) Diagnosing attention-deficit hyperactivity disorder (ADHD) in children involved with child protection services: are current diagnostic guidelines acceptable for vulnerable populations? Child Care Health Dev 41(2):178–185 24942100 10.1111/cch.12168

[9] Kääriälä A, Gyllenberg D, Sund R, Pekkarinen E, Keski-Säntti M, Ristikari T et al (2022) The association between treated psychiatric and neurodevelopmental disorders and out-of-home care among Finnish children born in 1997. Eur Child Adolesc Psychiatry 31(11):1789–1798 34101021 10.1007/s00787-021-01819-1 PMC9666323

[10] Jozefiak T, Kayed NS, Rimehaug T, Wormdal AK, Brubakk AM, Wichstrøm L (2016) Prevalence and comorbidity of mental disorders among adolescents living in residential youth care. Eur Child Adolesc Psychiatry 25(1):33–47 25749933 10.1007/s00787-015-0700-x PMC4698296

[11] Bronsard G, Alessandrini M, Fond G, Loundou A, Auquier P, Tordjman S et al (2016) The prevalence of Mental disorders among children and adolescents in the child Welfare System: a systematic review and Meta-analysis. Med (Baltim) 95(7):e2622 10.1097/MD.0000000000002622 PMC4998603 26886603

[12] Deutsch SA, Lynch A, Zlotnik S, Matone M, Kreider A, Noonan K (2015) Mental Health, behavioral and developmental issues for Youth in Foster Care. Curr Probl Pediatr Adolesc Health Care 45(10):292–297 26409926 10.1016/j.cppeds.2015.08.003

[13] Petrowski N, Cappa C, Gross P (2017) Estimating the number of children in formal alternative care: challenges and results. Child Abuse Negl 70:388–398 28578826 10.1016/j.chiabu.2016.11.026

[14] Eurochild-Better Data for Better Child Protection Systems in Europe [Internet]. [cited 2023 May 12]. Available from: https://www.eurochild.org/resource/better-data-for-better-child-protection-systems-in-europe/

[15] Simkiss DE, Spencer NJ, Stallard N, Thorogood M (2012) Health service use in families where children enter public care: a nested case control study using the General Practice Research Database. BMC Health Serv Res 12:65 22424404 10.1186/1472-6963-12-65 PMC3361673

[16] Brännström L, Vinnerljung B, Hjern A (2020) Outcomes in Adulthood after Long-Term Foster Care: a Sibling Approach. Child Maltreat 25(4):383–392 31960707 10.1177/1077559519898755

[17] Green MJ, Kariuki M, Chilvers M, Butler M, Katz I, Burke S et al (2019) Inter-agency indicators of out-of-home-care placement by age 13–14 years: a population record linkage study. Child Abuse Negl 93:91–102 31075574 10.1016/j.chiabu.2019.04.013

[18] Heino T, Hyry S, Ikäheimo S, Kuronen M, Rajala R (2016) Lasten kodin ulkopuolelle sijoittamisen syyt, taustat, palvelut ja kustannukset: HuosTa-hankkeen (2014–2015) päätulokset [Internet]. THL; [cited 2023 May 12]. Available from: https://www.julkari.fi/handle/10024/130536

[19] Ristikari T, Keski-Säntti M, Sutela E, Haapakorva P, Kiilakoski T, Pekkarinen E et al (2018) Suomi lasten kasvuympäristönä: Kahdeksantoista vuoden seuranta vuonna 1997 syntyneistä [Internet]. THL; [cited 2023 May 12]. Available from: https://www.julkari.fi/handle/10024/137104

[20] Russell J, Macgill S (2015) Demographics, policy, and foster care rates; a Predictive Analytics Approach. Child Youth Serv Rev 58:118–126

[21] Franzén E, Vinnerljung B, Hjern A (2008) The epidemiology of out-of-home care for children and youth: a national cohort study. Br J Soc Work 38(6):1043–1059

[22] Agnafors S, Torgerson J, Rusner M, Kjellström AN (2020) Injuries in children and adolescents with psychiatric disorders. BMC Public Health 20(1):1273 32838787 10.1186/s12889-020-09283-3 PMC7445910

[23] Simkiss DE, Stallard N, Thorogood M (2013) A systematic literature review of the risk factors associated with children entering public care. Child Care Health Dev 39(5):628–642 23210455 10.1111/cch.12010

[24] Zou G (2013) A modified Poisson regression approach to prospective studies with binary data. Am J Epidemiol 159(7):702–706 10.1093/aje/kwh090 15033648

[25] R: The R Project for Statistical Computing [Internet]. [cited 2023 May 12]. Available from: https://www.r-project.org/

[26] Lamsal, Zwicker R (2017) Economic evaluation of interventions for children with neurodevelopmental disorders: opportunities and challenges. Appl Health Econ Health Policy 15(6):763–772 10.1007/s40258-017-0343-9 PMC5701958 28822113

[27] Peverill, Dirks M, Narvaja MA, Herts T, Comer KL, McLaughlin JS (2021) Socioeconomic status and child psychopathology in the United States: a meta-analysis of population-based studies. Clin Psychol Rev 83:101933 10.1016/j.cpr.2020.101933 PMC7855901 33278703

[28] Keilow M, Wu C, Obel C (2020) Cumulative social disadvantage and risk of attention deficit hyperactivity disorder: Results from a nationwide cohort study. SSM - Popul Health. ;10:100548 10.1016/j.ssmph.2020.100548 PMC7016018 32072007

[29] Hall, Goldfeld T, Loftus S, Honisett H, Liu S, De Souza H (2022) Integrated Child and Family Hub models for detecting and responding to family adversity: protocol for a mixed-methods evaluation in two sites. BMJ Open 12(5):e055431 10.1136/bmjopen-2021-055431 PMC9125738 35613800

[30] Honisett S, Loftus H, Hall T, Sahle B, Hiscock H, Goldfeld S (2022) Do Integrated Hub Models of Care Improve Mental Health Outcomes for Children Experiencing Adversity? A Systematic Review. Int J Integr Care. ;22(2):24 10.5334/ijic.6425 PMC9205372 35756336

[31] Yonek J, Lee CM, Harrison A, Mangurian C, Tolou-Shams M (2020) Key Components of Effective Pediatric Integrated Mental Health Care Models: A Systematic Review. JAMA Pediatr. ;174(5):487–98 10.1001/jamapediatrics.2020.0023 PMC7483725 32150257

[32] Kylmäluoma A. Lastenpsykiatriseen osastohoitoon päätyneen lapsen polku sekä avun ja tuen tarpeiden tulkitseminen palvelujärjestelmässä [Internet]. Itä-Suomen yliopisto; 2021 [cited 2023 May 4]. Available from: https://erepo.uef.fi/handle/123456789/25466

[33] Takalo T, Räsänen S, Hakko H, Juutinen A, Niemelä M (2022) Rationale and Description of Implementation of Regional Collaborative Service Model for Enhancing Psychosocial Wellbeing of Children and Families-Oulu Collective Impact Study. Front Psychiatry. ;13:784995 10.3389/fpsyt.2022.784995 PMC8936173 35321229

[34] MacDonald, Turner GM (2007) Treatment Foster Care for improving outcomes in children and Young people. Campbell Syst Rev 3(1):1–95 10.1002/14651858.CD005649.pub2 18254087

[35] Åström T, Bergström M, Håkansson K, Jonsson AK, Munthe C, Wirtberg I et al (2020) Treatment Foster Care Oregon for Delinquent Adolescents: A Systematic Review and Meta-Analysis. Res Soc Work Pract. ;30(4):355–67

[36] Zeanah CH, Finelli J, Gleason MM (2019) Effective mental health treatment for children in foster care. Lancet Child Adolesc Health. ;3(3):136–7 10.1016/S2352-4642(19)30006-9 30704872

[37] Cuffe SP, Visser SN, Holbrook JR, Danielson ML, Geryk LL, Wolraich ML et al (2020) ADHD and Psychiatric Comorbidity: Functional Outcomes in a School-Based Sample of Children. J Atten Disord. ;24(9):1345–54 10.1177/1087054715613437 PMC4879105 26610741

[38] Liu, Huang CY, Kao WL, Gau WC (2017) Influence of disruptive Behavior disorders on Academic Performance and School functions of youths with Attention-Deficit/Hyperactivity disorder. Child Psychiatry Hum Dev 48(6):870–880 28168530 10.1007/s10578-017-0710-7

[39] Sund R (2012) Quality of the Finnish Hospital Discharge Register: a systematic review. Scand J Public Health. ;40(6):505–15 10.1177/1403494812456637 22899561

[40] Miettunen, Suvisaari J, Haukka J, Isohanni J (2011) M. Use of Register Data for Psychiatric Epidemiology in the Nordic Countries. In: Textbook of Psychiatric Epidemiology [Internet]. John Wiley & Sons, Ltd; [cited 2023 May 12]. p. 117–31. Available from: https://onlinelibrary.wiley.com/doi/abs/10.1002/9780470976739.ch8

[41] Kaiser NM, McBurnett K, Pfiffner LJ (2011) Child ADHD severity and positive and negative parenting as predictors of child social functioning: evaluation of three theoretical models. J Atten Disord. ;15(3):193–203 10.1177/1087054709356171 20424006

