# Supplementary material for: Child- and parent-related determinants for out-of-home care in a nationwide population with neurodevelopmental disorders: a register-based Finnish birth cohort 1997 study
Source: Eur Child Adolesc Psychiatry. 2024 Mar 2;33(10):3459–70. doi: 10.1007/s00787-024-02406-w (PMC11564338; doi:10.1007/s00787-024-02406-w)
Supplement: Supplementary file 1 — Supplementary Material 1 [file 787_2024_2406_MOESM1_ESM.docx]

**Supplemental materials**

Appendix A

**Table A1. Statistics of out-of-home care group**

| **Statistics of out-of-home care group (N=903)** |  |
| --- | --- |
| **Mean age at first placement (Years, sd)** | 10.3 (5.6) |
| **Mean age at first NDD diagnosis (Years, sd)** | 9.5 (4.6) |
| **First NDD diagnosis before the first placement (%)** | 52.6% |
| **Mean time interval between the first NDD diagnosis and the first placement given that the NDD diagnosis preceded placement (Years, sd)** | 5.5 (3.8) |
| **Mean time interval between the first placement and the first NDD diagnosis given that the placement preceded NDD diagnosis (Years, sd)** | 4.3 (3.9) |
| **Duration of placement (summed, years) (Mean, sd)** | 4.08 (4.8) |
| **Periods in OHC (Mean, sd)** | 4.12 (4.53) |
| Age distribution, the first placement in OHC, n (%)  0-1 years  2-12 years  13-17 years | 120 (13.29%)  380 (42.08%)  403 (44.63%) |
| **Gender at birth, boy, n (%)** | 591 (65.44%) |
| **Gender at birth, girl, n (%)** | 312 (34.55%) |
| **Legal grounds of the placement decision according to the most common ground, n (%)**  Short-term placement as a support measure in open care  Taken into care  Emergency placement of the child  Taken into care involuntarily | 285 (31.56%)  405 (44.85%)  117 (12.96%)  96 (10.63%) |
| **Placement setting, according to the longest period setting**  Residential care  Professional foster home  Foster family  Other | 536 (59.36%)  155 (17.17%)  176 (19.49%)  36 (3.99%) |

**Table A2** ICD10 codes for external causes for hospitalisations

| Assaults  Poisonings  Accidents  Injuries | X85, X90-X99, Y00-Y09  X40-X49, T36-39, T45, T60-T65  V00-V99, W00-W99, X10-X39, X50-X59  S00-S99, T00-T35, T66-T78 |
| --- | --- |
